# Single‐cell RNA sequencing reveals SERPINE1‐expressing CAFs remodelling tumour microenvironment in recurrent osteosarcoma

**DOI:** 10.1002/ctm2.1527

**Published:** 2024-01-09

**Authors:** Xin Huang, Lutong Wang, Haoyu Guo, Weiyue Zhang

**Affiliations:** ^1^ Department of Orthopaedics Union Hospital, Tongji Medical College, Huazhong University of Science and Technology Wuhan China; ^2^ Department of Endocrinology Union Hospital, Tongji Medical College, Huazhong University of Science and Technology Wuhan China

Dear Editor,

Osteosarcoma (OS) is widely regarded as the most common bone tumour, mainly affecting young adults.^1^ Cancer‐associated fibroblasts (CAFs), which are highly infiltrated in the tumour microenvironment (TME), could act as a novel target for cancer immunotherapy.^2^, ^3^ Single‐cell RNA sequencing (scRNA‐seq) has been regarded as one potent new method to investigate TME. We aim to uncover the roles of CAFs in recurrent OS with scRNA‐seq and further provided novel treatment strategies for OS.

Our study conducted scRNA‐seq and bioinformatic analysis to uncover cellular clusters of OS tumour tissues. The cellular clusters of OS lesions were explored via two Gene Expression Omnibus (GEO) datasets of GSE152048 (BC) and GSE162454 (OS). Moreover, three paired OS lesions were utilised to further confirm the outcomes. After quality control, these two datasets were grouped into 14 cell clusters with the Uniform Manifold Approximation and Projection (UMAP) method according to the corresponding biomarkers (Figure 1A). Div‐cells are the MKI67+ diverse cells with improved proliferation. We identified the cells in TME including: OS cells (COL1A1+, RUNX2+, ALPL+), Div‐OS cells (COL1A1+, RUNX2+, ALPL+; TOP2A+, MKI67+), CAFs (ACTA2+/α‐smooth muscle actin (α‐SMA), COL1A1+, FGF7+), Div‐CAFs (ACTA2+/α‐SMA, COL1A1+, TOP2A+, FGF7+, MKI67+), macrophages (CD74+, CD14+, C1QA+, C1QB+) and so on. The UMAP plots of cell clusters (Figure 1B) and the relative proportion of every cluster (Figure 1C) in these two datasets were shown. Recurrent samples (BC11 and BC20) had a higher infiltrate of Div‐CAFs than other samples (Figure S1A). As for the ‘metastasis’ and ‘recurrent’ samples, ‘metastasis’ refers to the samples collected from the lung metastatic OS lesions, and ‘recurrent’ refers to the samples collected from recurrent OS lesions. Based on the above cell clusters in different OS tissues shown via the UMAP plots (Figure 1D), we found that CAFs were more highly infiltrated in recurrent OS than the primary OS (96.42% vs. 3.58%). The Differentially expressed genes (DEGs) among different OS tissues were further studied. Based on the pathway enrichment analysis, we found the significantly activated the epithelial‐to‐mesenchymal transition (EMT) pathway in recurrent OS (Figure 1E, shown by green arrow), which showed the heterogeneity of gene expressions among different OS tissues. Furthermore, the density heatmap and scatterplot of Gene Set Enrichment Analysis (GSEA) indicated that CAFs were remarkably associated with EMT hallmark gene sets (Figure 1F,G). As shown by Figure 1G, EMT sets have the maximum density in the cell cluster of CAFs. To sum up, CAFs were highly infiltrated and associated with the EMT in recurrent OS.

**FIGURE 1:**
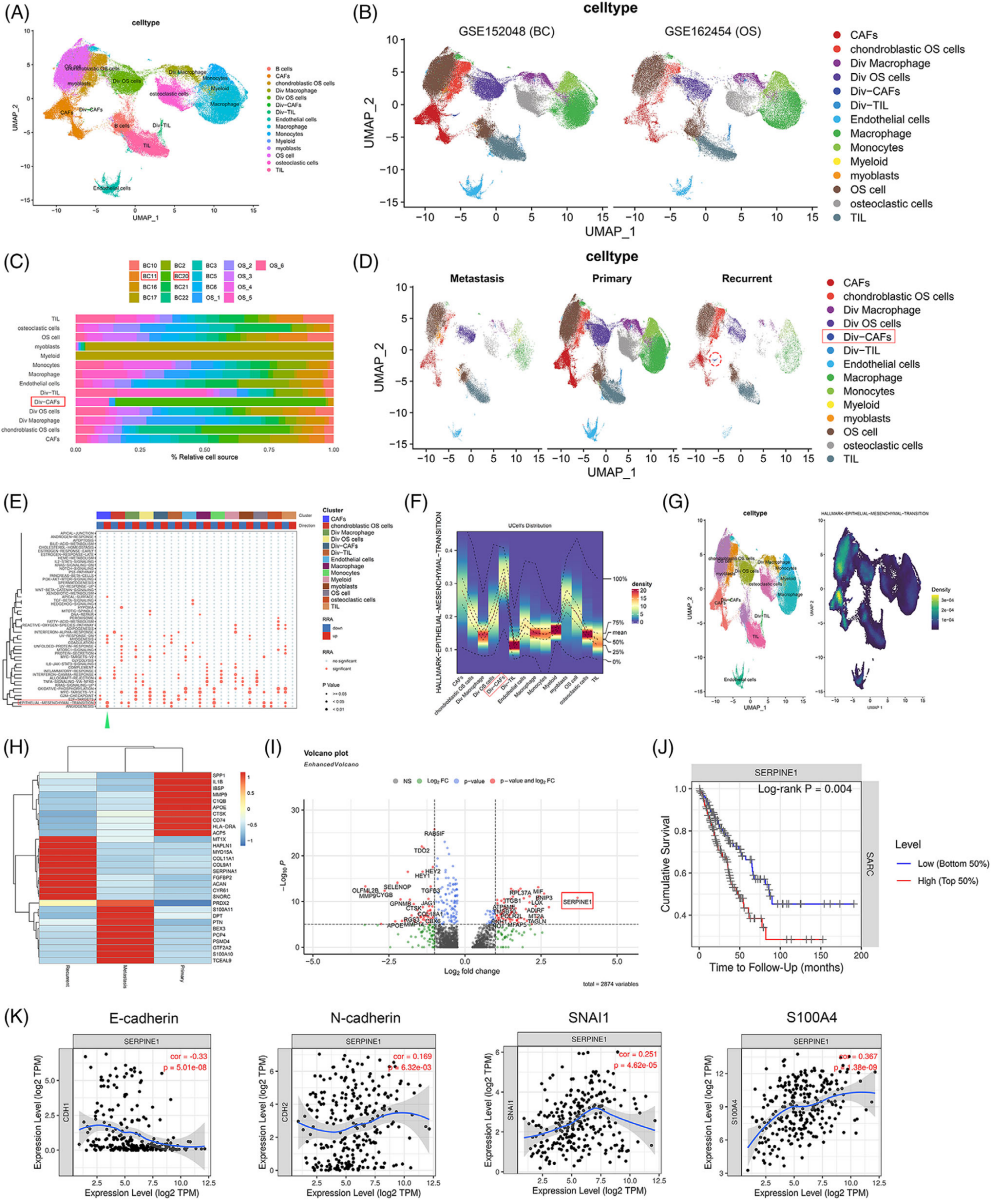
The cellular clusters of osteosarcoma (OS) tissues and clinical characterisations of SERPINE1‐expressing cancer‐associated fibroblasts (CAFs) in recurrent OS. (A) The Uniform Manifold Approximation and Projection (UMAP) plots of 14 cell clusters in OS tissues. (B) The UMAP plots of 14 cell clusters in the two datasets. (C) The relative proportion of every cluster in the two datasets. (D) The UMAP plots of cell clusters in different OS tissues. (E) The heatmap of GSEA of the 50 hallmark gene sets in the 14 cell clusters. (F) The density heatmap and (G) the scatterplot of GSEA of epithelial‐to‐mesenchymal transition (EMT) hallmark genes in cell clusters. (H) The DEGs in different OS tissues. (I) The volcano plot showed DEGs of Div‐CAFs. (J) Cumulative survival curves of SERPINE1 in sarcoma. (K) The correlations between SERPINE1 and EMT markers.

The DEGs in different OS tissues were shown (Figure 1H). Moreover, SERPINE1 was significantly higher in Div‐CAFs of recurrent OS (Figure 1I). SERPINE1 is also termed plasminogen activator inhibitor 1 (PAI1), and its encoded protein inhibits the fibrinolysis process. A previous study has shown that SERPINE1 functions as a target gene of microRNA to promote the invasion and metastasis of OS.^4^ SERPINE1 has been reported as a pro‐tumourigenesis factor and a member of the EMT way, which suggested that SERPINE1 enables tumour cell growth and invasion.^5^ Furthermore, we tried to identify the clinical characterisations of SERPINE1‐expressing CAFs. Cumulative survival curves indicated that SERPINE1 was associated with a poor prognosis of sarcoma (Figure 1J). Moreover, SERPINE1 was significantly associated with the EMT markers (Figure 1K). Accordingly, SERPINE1 might contribute to the EMT of OS, thereby serving as a novel biomarker for recurrent OS.

To validate the findings above, we explored the functions of CAFs in recurrent OS. By TIMER database, fibroblast activation protein (FAP) of CAFs was significantly associated with EMT markers (Figure S1B). By western blot, increased N‐cadherin or decreased E‐cadherin expression was shown in CAFs rather than OS cells (Figure S1C,D). As shown by immunofluorescence (IF) staining, the increased α‐SMA and decreased E‐cadherin expressions were shown in recurrent OS (Figure S1E). To sum up, it was indicated that CAFs were remarkably associated with EMT markers in recurrent OS.

The functions of SERPINE1 in vivo were further studied. Nude mice (BALB/c‐nu, female, 4−5 weeks) were injected subcutaneously with 5 × 10^6^ MNNG/HOS cells. The tumour tissues were further treated by the injection of si‐SERPINE1 or si‐NC. Compared with si‐NC group, si‐SERPINE1 remarkably inhibited the tumour volume (Figure 2A). SERPINE1 was remarkably downregulated in si‐SERPINE1 group via Immunohistochemistry (IHC) (Figure 2B,E). The Ki67+ cells significantly decreased (Figure 2C,E) but TUNEL positive cells increased (Figure 2D,E) because of si‐SERPINE1. The above results suggested that SERPINE1 knockdown has significantly anti‐OS effects. IF staining indicated that the relative proportion of CAFs was significantly lower in si‐SERPINE1 group (Figure 2F). We found that IL1B of Div‐Macrophages was significantly downregulated (Figure 2G). SERPINE1 has a positive correlation with CD163 (*r* = .443, *p* = 0e‐00), which is the marker of M2 macrophages (Figure 2H). Moreover, in si‐SERPINE1, M1 macrophages (iNOS) remarkably increased (Figure 2I). Therefore, SERPINE1 could regulate macrophage polarisation and inhibit OS progression in vivo.

**FIGURE 2:**
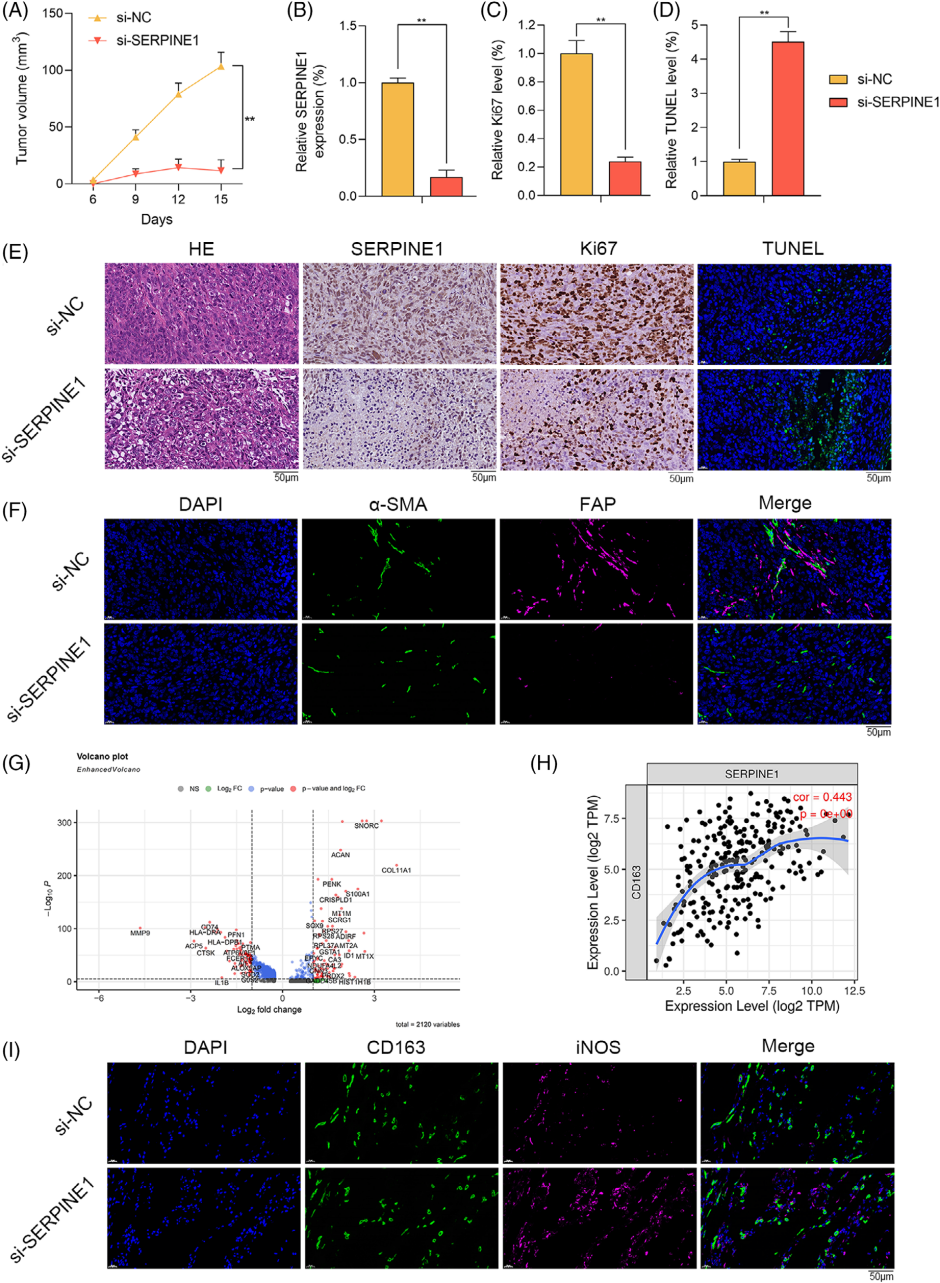
The effects of SERPINE1 knockdown on osteosarcoma (OS) in vivo. (A) The volumes of subcutaneously transplanted tumours in si‐NC and si‐SERPINE1 groups (^**^ *p* < .01). (B and E) The expression of SERPINE1 in tumours via IHC staining (scale bar: 50 μm; ^**^ *p* < .01). (C and E) Ki67 staining indicated the proliferation rates of tumour cells (scale bar: 50 μm; ^**^ *p* < .01). (D and E) TUNEL staining indicated the apoptotic rates of tumour cells (scale bar: 50 μm; ^**^ *p* < .01). (F) The relative proportion of cancer‐associated fibroblasts (CAFs) via immunofluorescence (IF) staining (scale bar: 50 μm). (G) The volcano plot indicated DEGs of Div‐Macrophages in OS. (H) The correlation between SERPINE1 and CD163 via TIMER database. (I) The relative proportion of macrophages via IF staining (scale bar: 50 μm).

As shown in Figure 3A, this study used the co‐culture systems to investigate the underlying mechanisms of SERPINE1 in the TME. CAFs transfected with si‐SERPINE1 had lower expression levels of the stromal activation markers including α‐SMA and FAP (Figure 3B,C). Accordingly, SERPINE1 is important in promoting the activity of CAFs. As for the lower chamber, OS cells in si‐SERPINE1 group showed decreased N‐cadherin and increased E‐cadherin expressions (Figure 3D). Furthermore, the relative proportion of M2 macrophages (CD163) in si‐SERPINE1 group remarkably reduced (Figure 3E,F). To sum up, SERPINE1 could regulate the activity of CAFs, further induce macrophage polarisation to facilitate OS recurrence.

**FIGURE 3:**
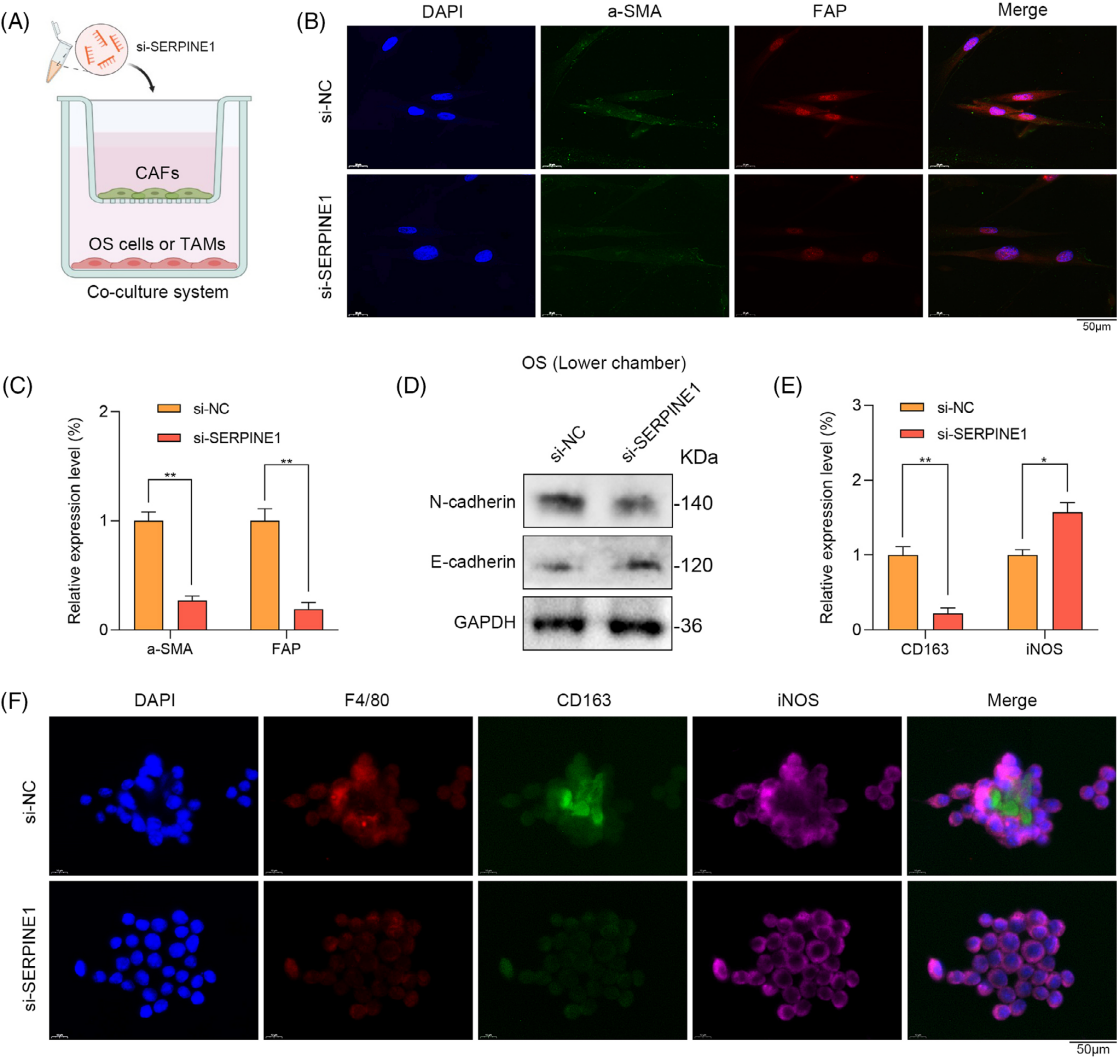
SERPINE1‐expressing cancer‐associated fibroblasts (CAFs) might remodel tumour microenvironment (TME) in recurrent osteosarcoma (OS). (A) The co‐culture system was utilised to detect the functions of SERPINE1 in the TME. (B and C) The α‐SMA and fibroblast activation protein (FAP) expressions of CAFs via immunofluorescence (IF) staining (scale bar: 50 μm; ^**^ *p* < .01). (D) Western blot showed the expressions of N‐cadherin and E‐cadherin. (E and F) The relative proportion of M2 macrophages (CD163) in si‐SERPINE1 group (scale bar: 50 μm; ^*^ *p* < .05) ^**^ *p* < .01.

The heterogeneity in TME among different tumours remains to be explored.^6^ The scRNA‐seq technology is widely used to identify the cellular groups in TME.^7^, ^8^ Compared with primary OS, we found that recurrent OS was featured by higher CAFs levels in TME via scRNA seq analysis. Moreover, the increased infiltrating CAFs were associated with the EMT in recurrent OS. Thus, this validated that CAFs could mediate OS recurrence. As for the detailed mechanisms, SERPINE1 had the highest expression in CAFs of recurrent OS. SERPINE1 has been widely known to regulate the progression of numerous cancers, which highlights the application of SERPINE1 as a potential target.^9^, ^10^ We validated that SERPINE1 was remarkably correlated with macrophage infiltrations and the EMT of OS. Knocking down SERPINE1 by si‐SERPINE1 could decrease CAFs infiltration and activity, and inhibit OS progression via inducing M1 macrophage polarisation (Figure 4).

**FIGURE 4:**
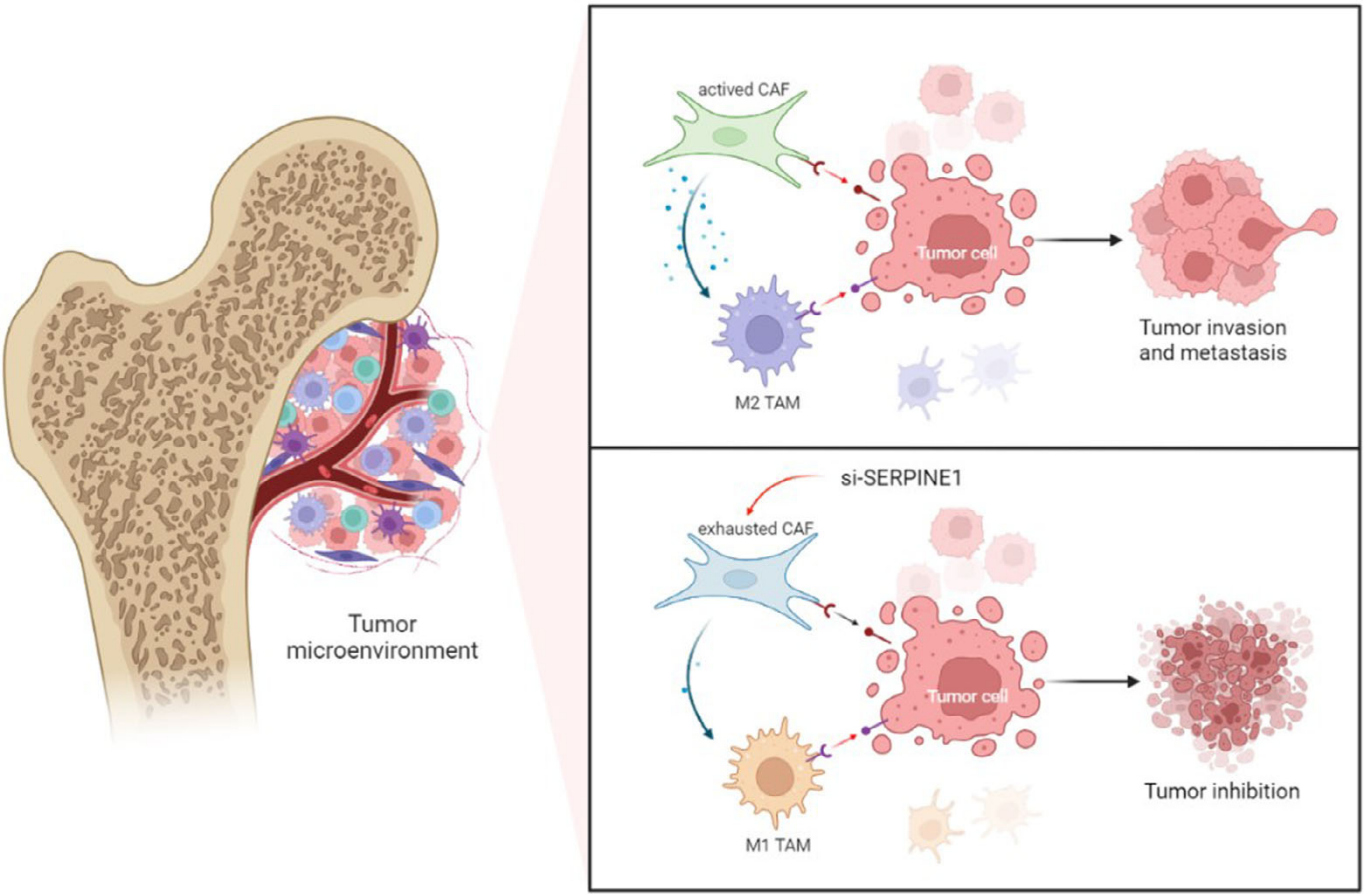
Targeting SERPINE1‐expressing cancer‐associated fibroblasts (CAFs) to remodel tumour microenvironment (TME) and treat recurrent osteosarcoma (OS).

To sum up, we uncovered the characteristics of recurrent OS and the roles of SERPINE1‐expressing CAFs in regulating TME. SERPINE1 could regulate the activity of CAFs, further induce macrophage polarisation and regulate EMT pathway to facilitate OS recurrence. Accordingly, targeting SERPINE1‐expressing CAFs had shown promising efficacy in treating recurrent OS. In the future, with the development of single‐cell multi‐omics technologies, we might precisely regulate the SERPINE1‐expressing CAFs in the TME, thereby contributing to the precise targeted therapy in OS.

## AUTHOR CONTRIBUTIONS

*Investigation, supervision and writing—original draft*: Xin Huang. *Investigation and methodology*: Haoyu Guo and Lutong Wang. *Conceptualisation, funding and writing—review and editing*: Xin Huang and Weiyue Zhang.

## CONFLICT OF INTEREST STATEMENT

The authors declare they have no conflicts of interest.

## ETHICS STATEMENT

This study was approved by the Institutional Animal Care and Use Committee of Huazhong University of Science and Technology (IACUC number 3068).

## Supporting information


**FIGURE S1** Cancer‐associated fibroblasts (CAFs) were associated with epithelial‐to‐mesenchymal transition (EMT) in recurrent osteosarcoma (OS). (A) The infiltration of Div‐CAFs in different samples. (B) The correlations between fibroblast activation protein (FAP) of CAFs and EMT markers of N‐cadherin and E‐cadherin. (C and D) The N‐cadherin and E‐cadherin expressions in CAFs and OS cells via western blot (^**^ *p* < .01). (E) The expressions of α‐SMA and E‐cadherin by immunofluorescence (IF) staining (scale bar: 100 μm; ^**^ *p* < .01).

## Data Availability

All data are available in the main text or the supplementary materials.
