# Supplementary figures and images for: Single‐cell RNA sequencing reveals SERPINE1‐expressing CAFs remodelling tumour microenvironment in recurrent osteosarcoma
Source: Clin Transl Med. 2024 Jan 9;14(1):e1527. doi: 10.1002/ctm2.1527 (PMC10775180; doi:10.1002/ctm2.1527)

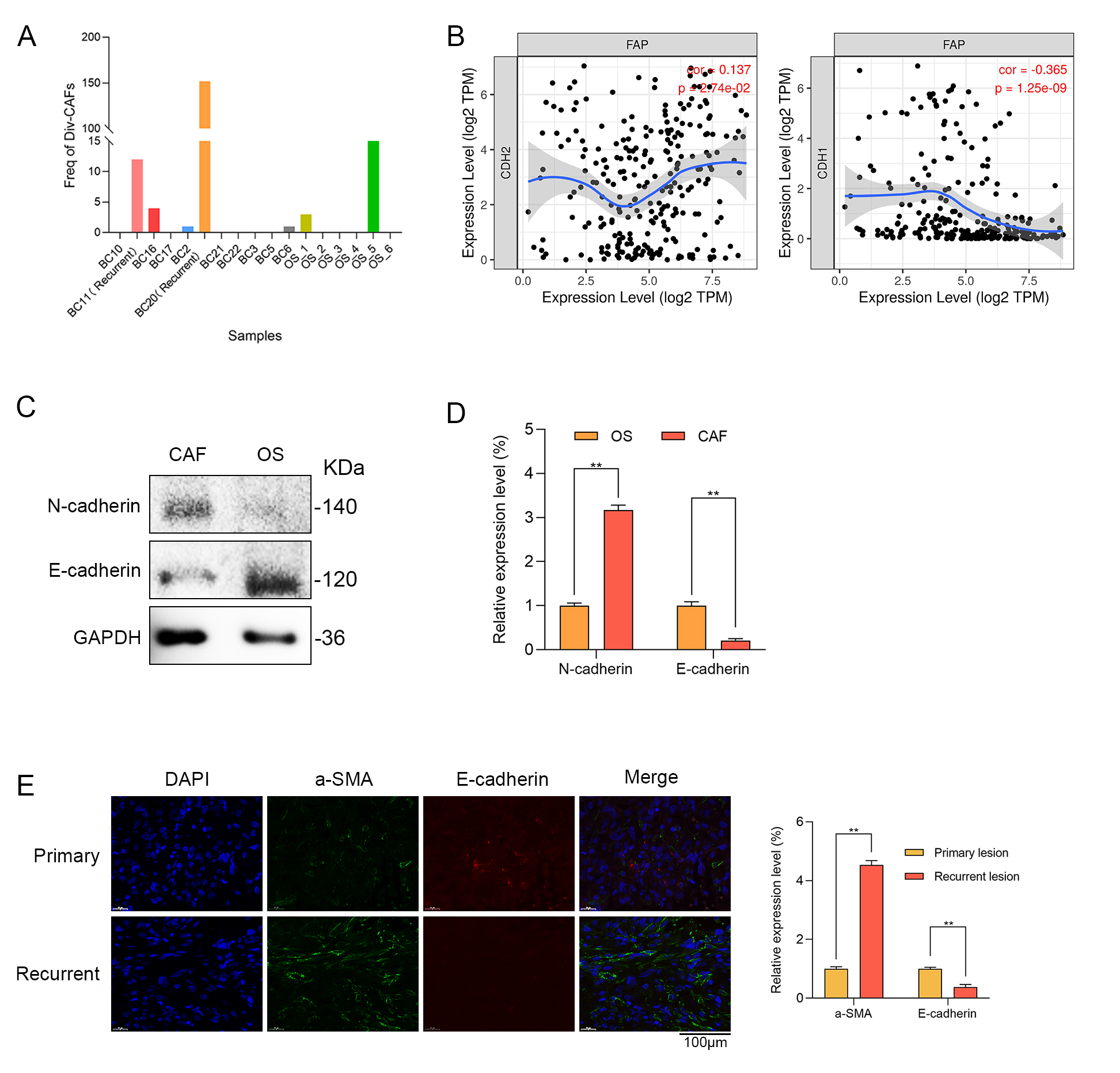

Supplement: Supplementary file 1 — FIGURE S1 Cancer‐associated fibroblasts (CAFs) were associated with epithelial‐to‐mesenchymal transition (EMT) in recurrent osteosarcoma (OS). (A) The infiltration of Div‐CAFs in different samples. (B) The correlations between fibroblast activation protein (FAP) of CAFs and EMT markers of N‐cadherin and E‐cadherin. (C and D) The N‐cadherin and E‐cadherin expressions in CAFs and OS cells via western blot (** p < .01). (E) The expressions of α‐SMA and E‐cadherin by immunofluorescence (IF) staining (scale bar: 100 μm; ** p < .01). [file CTM2-14-e1527-s001.tif]
